# An Unusual Long-Term Complication of Bariatric Surgery

**DOI:** 10.7759/cureus.23931

**Published:** 2022-04-07

**Authors:** Cassandra M Voong, Kenneth J Vega, Ahmad Alkaddour

**Affiliations:** 1 Internal Medicine, Medical College of Georgia - Augusta University, Augusta, USA; 2 Gastroenterology and Hepatology, Medical College of Georgia - Augusta University, Augusta, USA

**Keywords:** laparoscopic roux-en-y gastric bypass, bariatric surgery complications, roux-en-y, liver failure, obesity

## Abstract

Roux-en-Y gastric bypass (RNYGB) has become the standard of care in treating obesity, a global health concern with associated comorbidities contributing to rising health care costs [1]. The positive outcomes of RNYGB have been well documented along with adverse effects such as nutrient deficiencies, hernia, postprandial dumping syndrome, chronic kidney disease and hypoglycemia. A lesser-known long term complication of RNYGB is liver failure. Here, we present a case where RNYGB performed 10 years prior contributed to acute liver failure.

## Introduction

Obesity is a pandemic that has been contributing to increased health care costs around the world due to the development of comorbidities [1] such as non-alcoholic fatty liver disease (NAFLD), diabetes, and cardiovascular diseases [2,3]. Recently, bariatric surgery has grown in popularity due to its effectiveness in attaining persistent weight loss [4]. Multiple investigators have shown bariatric surgery drastically decreases weight and body mass index (BMI) within the first year, along with eliminating the comorbidities mentioned above through improved glycemic and lipid control [1,5]. Despite the benefits of bariatric surgery, postoperative complications occur. These more commonly include nutrient deficiencies (vitamin B1, B12, iron, calcium), hernia, postprandial dumping syndrome, chronic kidney disease, and hypoglycemia [6]. A rare complication post-Roux-en-Y gastric bypass (RNYGB) is liver failure [4]. We present a case where RNYGB performed 10 years prior contributed to acute liver failure.

## Case presentation

A 30-year-old female presented to the emergency department (ED) with a rapid onset of abdominal pain along with swelling and bilateral lower extremity edema that progressed over three days. Her past medical history consists of GERD s/p laparoscopic Nissen fundoplication at age 15 and later RNYGB (10 years ago at another hospital), endometrioses, anxiety, and depression.

In the ED, her heart rate was in the low 100s beats per minute, respiratory rate of 18 breaths per minute, blood pressure of 136/79 mm Hg, and her O2 saturation was normal on room air. Her blood work was significant for leukocytosis of 30,000 cmm with a left shift, hemoglobin 8.7 g/dl (was 14 g/dl in 2006), INR 1.5, sodium 129 mmol/L, bicarbonate 18 mEq/L, and pH of 7.44 on a venous blood gas. Her physical exam was remarkable for conjunctival jaundice and 4+ lower extremity pitting edema up to her lower abdomen. A CT of the abdomen and pelvis obtained in the ED showed evidence of portal hypertension and nodular liver with steatosis as well as patchy airspace opacities throughout bilateral lungs that were concerning for atypical viral pneumonia. Further evaluation with an abdominal ultrasound suggested findings that were consistent with cirrhosis and portal hypertension with no evidence of thrombosis and small volume ascites. The patient has no previous diagnosis of cirrhosis, however, she disclosed a drinking history. She was a daily drinker for several years in her early 20s, consuming three to four malt beverages daily, and currently consuming mixed cocktails and malt beer every other day (on average three to four nights per week). Her last drink was a month before admission.

Her usual body weight was 80 kg (height 158 cm). She endorsed a 48 lbs unintentional weight loss over two months and presented at 60 kg on admission. The patient’s family noticed the patient became deconditioned and malnourished two years prior. Her post-operative complications involved malabsorption with intractable nausea and vomiting, requiring multiple ED visits. A prior CT of the abdomen in 2018 showed normal liver. Furthermore, a recent gastric emptying test performed showed “rapid gastric emptying.” Upon further questioning, her nutrition was further compromised with medication noncompliance of recommended vitamins and minerals due to lack of insurance and was lost to follow up after her RNYGB procedure.

During her hospitalization, her anasarca progressed as her acute on chronic decompensated cirrhosis worsened with her MELD score reaching a peak of 27, INR 2.1 and Cr from 0.5 mg/dl to 2.95 mg/dl within eight days. She then developed acute hypoxic respiratory failure and was placed on mechanical ventilation in the ICU. Her kidney function continued to deteriorate likely due to hepatorenal syndrome and was transferred to a liver transplant center. Unfortunately, she was not a candidate for transplant as she was deemed too unstable and suggested to re-evaluate outpatient once the patient is extubated. A liver biopsy was deferred as well due to her unstable condition. After a prolonged hospital stay at the transplant center, the patient was transferred back with the etiology of her hepatic failure remaining unknown despite ruling out other chronic liver diseases and acute toxins. The transplant center asserted it was unlikely that alcoholic cirrhosis contributed to her acute condition. Consequently, we suspect post-bariatric surgery liver failure as a culprit.

## Discussion

The RNYGB procedure consists of the creation of a Roux-en-Y gastrojejunostomy along with a gastric pouch [6]. This surgery results in caloric intake restriction, delivers nutrients to the small bowel rapidly, while increasing bile/nutrient delivery to the distal portion of the small intestine and concomitantly excluding nutrients to the proximal portion [6]. The process can precipitate significant weight loss and place metabolic syndrome into remission [7]. The mean excess weight loss (EWL) post-operatively in the first year was 80.4% and 74.8% five years later after an RNYGB [8]. Our patient’s RNYGB was performed at an external hospital and was lost to follow-up for many years, hence her immediate EWL post-surgically is unknown. 

Despite the positive effects of RNYGB, reports of liver injury are rare. Three mechanisms can explain liver injury induced by bariatric surgery, which include drastic/rapid weight loss, caloric-protein malnutrition plus bacterial overgrowth and alteration of gut microbiota [9].

Rapid weight loss and/or malnutrition have been shown to exacerbate steatohepatitis and induce inflammation in the liver with the development of liver failure in a fairly short period [10]. Our patient had 48 lb weight loss over two months and underlying steatosis although it is unknown whether she had nonalcoholic steatohepatitis (NASH). The pathophysiology is still not well understood [9]. Malnutrition is characterized by edema, hypoalbuminemia, asthenia, and anemia, which were all experienced by our patient [9]. It is suggested that malabsorption can lead to malnutrition, which can cause hypoalbuminemia, facilitating NASH directly. Subsequently, portal hypertension can arise creating a vicious cycle as it can subsequently decrease intestinal absorption [9].

Other complications from RNYGB include calcium and iron deficiency, vitamin B1 and B12 deficiency, and post-prandial dumping syndrome [6]. This can all be corrected with surgical reversal if medical management fails. Although malnutrition is a rare complication of RNYGB, it is recommended to have high protein/vitamin/mineral supplements with frequent liver enzyme surveillance [9]. Interestingly, one systematic review of liver failure after RNYGB by Mahawar et al. does not recommend routine monitoring of liver function tests (LFTs) after every bariatric surgery due to the very low incidences of liver failure. However, they did suggest routine monitoring should be performed on those with known cirrhosis, abuse of alcohol, a new illness, surgery-related complications and RNYGB that had undergone longer limb variants. Monitoring LFTs in these populations may assist in diagnosing those with severe malnutrition post-operatively from RNYGB [4]. Our patient would have benefited from routine LFTs given her reported history of alcohol consumption. Unfortunately, these recommendations were not performed on the patient due to medication non-adherence and loss of follow-up.

Bacterial overgrowth induces inflammation in the liver by activating Kupffer cells to release tumor necrosis factor-alpha (TNF-α) in response to the bacterial lipopolysaccharides and ethanol produced by the bacteria [2]. There is increased gut permeability in the small intestine with bacterial overgrowth in those with NASH compared to their counterparts without NASH. This may explain liver fibrosis and rapid onset of NASH in those with a jejunoileal bypass such as our patient [1]. Although malnutrition is a rare complication of RNYGB, high protein/vitamin/mineral supplementation is recommended [9]. One systematic review of liver failure after RNYGB did not recommend routine monitoring of LFTs after bariatric surgery due to the very low incidence of liver failure [4]. However, monitoring was suggested for those with known cirrhosis, actively abusing alcohol, a newly diagnosed liver-related illness, surgery-related complications, or RNYGB with longer limb variants [4].

## Conclusions

In conclusion, postoperative complications of RNYGB such as rapid weight loss and malnutrition could contribute to liver failure as late as a decade later. Our patient had evidence of steatosis and portal hypertension. However, her rapid decline in kidney and liver function without sepsis makes post-bariatric liver failure a culprit. This is an important clinical entity for clinicians to be aware of when acute liver failure occurs in a bariatric patient. Studies are needed in the bariatric population to completely understand this relationship.
